# Exploring the shared genetic landscape of diabetes and cardiovascular disease: findings and future implications

**DOI:** 10.1007/s00125-025-06403-9

**Published:** 2025-03-15

**Authors:** Hyunsuk Lee, Maria Fernandes, Jeongeun Lee, Jordi Merino, Soo Heon Kwak

**Affiliations:** 1 Department of Internal Medicine, Seoul National University College of Medicine and Seoul National University Hospital, Seoul, Korea; 2 Department of Translational Medicine, Seoul National University College of Medicine, Seoul, Korea; 3 Genomic Medicine Institute, Medical Research Center, Seoul National University College of Medicine, Seoul, Korea; 4 Novo Nordisk Foundation Center for Basic Metabolic Research, University of Copenhagen, Copenhagen, Denmark; 5 Diabetes Unit, Endocrine Division, Massachusetts General Hospital, Boston, MA USA; 6 Center for Genomic Medicine, Massachusetts General Hospital, Boston, MA USA

**Keywords:** Cardiovascular disease, Coronary artery disease, CVD, Genetics, Review, Type 2 diabetes

## Abstract

Diabetes is a rapidly growing global health concern projected to affect one in eight adults by 2045, which translates to roughly 783 million people. The profound metabolic alterations often present in dysglycaemia significantly increase the risk of cardiovascular complications. While genetic susceptibility plays a crucial role in diabetes and its vascular complications, identifying genes and molecular mechanisms that influence both diseases simultaneously has proven challenging. A key reason for this challenge is the pathophysiological heterogeneity underlying these diseases, with multiple processes contributing to different forms of diabetes and specific cardiovascular complications. This molecular heterogeneity has limited the effectiveness of large-scale genome-wide association studies (GWAS) in identifying shared underlying mechanisms. Additionally, our limited knowledge of the causal genes, cell types and disease-relevant states through which GWAS signals operate has hindered the discovery of common molecular pathways. This review highlights recent advances in genetic epidemiology, including studies of causal associations that have uncovered genetic and molecular factors influencing both dysglycaemia and cardiovascular complications. We explore how disease subtyping approaches can be critical in pinpointing the unique molecular signatures underlying both diabetes and cardiovascular complications. Finally, we address critical research gaps and future opportunities to advance our understanding of both diseases and translate these discoveries into tangible benefits for patient care and population health.

**Graphical Abstract:**

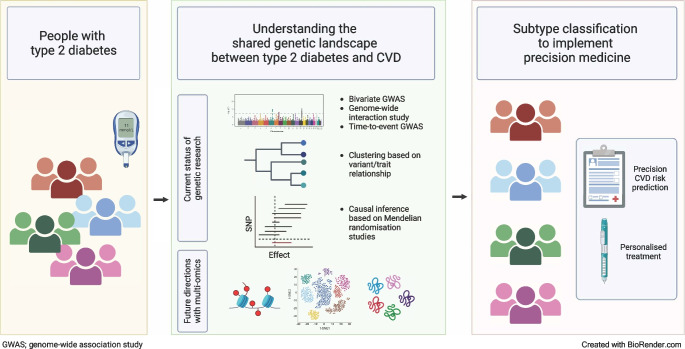

**Supplementary Information:**

The online version contains a slideset of the figures for download available at 10.1007/s00125-025-06403-9.

## Introduction

Type 2 diabetes significantly elevates the risk of CVD, the leading cause of mortality globally [1, 2]. This ‘double threat’ is not a coincidence. Over a century of research has established a strong link between type 2 diabetes and CVD. Early autopsy studies revealed accelerated atherosclerosis in individuals with diabetes, and subsequent epidemiological studies consistently demonstrated a two- to threefold increased risk of cardiovascular complications in those with diabetes [3–5]. Additionally, CVD in people with type 2 diabetes presents with distinct clinical features, such as earlier onset, multi-vessel involvement and longer diseased vessel segments [4]. Although the event rate of CVD in people with type 2 diabetes has decreased over the past few decades [5], the risk remains elevated compared with that in the general population [6, 7].

Individuals with type 2 diabetes or even with impaired glucose tolerance share common risk factors for CVD, including elevated LDL-cholesterol (LDL-C), high BP, obesity and smoking, reflecting the largely similar underlying drivers of atherosclerosis. However, dysglycaemia introduces an additional layer of complexity. The metabolic derangements underlying dysglycaemia, which encompasses a particular lipid profile characterised by atherogenic lipoproteins, chronic low-grade inflammation and oxidative stress, promote endothelial dysfunction and accelerate atherogenesis. This shared and exacerbated pathophysiology is the foundation of the ‘common soil’ hypothesis [8], which postulates that both dysglycaemia and atherosclerosis arise from a common pool of genetic and environmental factors influencing disease initiation, progression and manifestation. While environmental factors such as smoking, diet, physical activity and air pollution are likely to play a role in both diseases, our understanding of the shared genetic links is less clear.

Modern human genetic epidemiology studies provide an attractive approach to elucidate the molecular underpinnings of the common soil hypothesis linking type 2 diabetes and CVD. However, most large-scale genome-wide association studies (GWAS) have focused on investigating the genetic risk of each disease separately, with only a limited number of studies conducted to identify overlapping genetic signals between the two [8]. Additionally, most previous studies have been cross-sectional, and longitudinal studies that clearly identify CVD events after type 2 diabetes diagnosis have been scarce. Moreover, the high aetiological heterogeneity observed in both type 2 diabetes and CVD reduces the likelihood of successfully identifying key genetic contributors to this overlap. A prime example of this heterogeneity is early-onset type 2 diabetes, in which the clinical presentation and underlying mechanisms differ significantly between youth/adolescents and adults. Notably, early-onset type 2 diabetes is often characterised by a rapid decline in beta cell function and a higher propensity for developing microvascular and macrovascular complications than later-onset disease [9–11]. Emerging subclassification schemas with refined subgroups have advanced our understanding of the heterogeneity of the pathophysiology of type 2 diabetes and its complications. Such approaches could lead to the discovery of shared genes and molecular processes, with the potential to identify more effective strategies to manage diabetes and its cardiovascular complications.

This review focuses on recent research identifying shared genetic risk loci between type 2 diabetes and CVD, providing an overview of key findings rather than a detailed technical discussion of the methods and statistical genetics tools used to uncover these loci. It also discusses disease-specific subtyping approaches, exploring how this evolving understanding can inform targeted prevention and management strategies to reduce the burden of multimorbidity in individuals with type 2 diabetes.

## Discovery of shared genetic regions between type 2 diabetes and CVD

Type 2 diabetes and CVD are complex diseases with an estimated heritability of 30–70% [12, 13]; thus, the potential influence of genetics on disease risk is significant. The most recent GWAS of type 2 diabetes identified more than 600 genomic loci associated with the disease [14]. At the same time, there is evidence of more than 200 genomic regions associated with coronary artery disease (CAD), 89 regions associated with stroke, and 19 regions associated with peripheral artery disease (PAD) [15–17]. The identification of such genetic variants is a critical initial step for advancing our understanding of the pathogenesis of type 2 diabetes and CVD. It also provides a unique opportunity to investigate if there are intermediate molecular processes that contribute to the development of both diseases (see Text box: Statistical methodologies and considerations for finding shared genetic loci between type 2 diabetes and CVD).


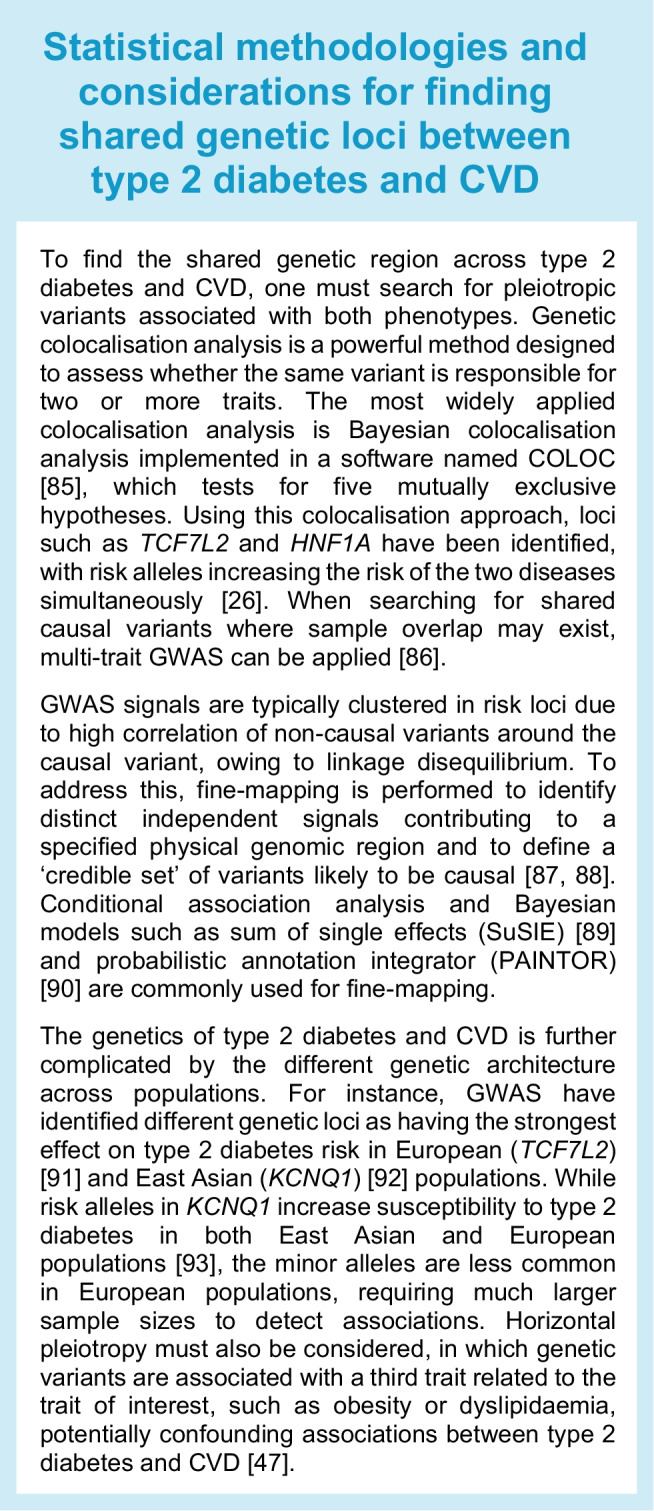


Previous studies have found a significant genetic correlation between type 2 diabetes and CVD entities such as CAD or stroke. For example, the genetic correlation between type 2 diabetes and CAD is around 0.3, meaning that there are multiple shared genetic regions between these diseases [18]. However, discovering shared genomic regions has proven challenging. Early attempts to identify shared signals have pointed to genes involved in insulin resistance pathways and identified genomic regions near the *IRS1* gene and on chromosome 9p21 near the *CDKN2A* and *CDKN2B* genes [8, 19, 20]. The *IRS1* gene plays a crucial role in insulin signalling and glucose homeostasis [21]. *CDKN2A* and *CDKN2B* are involved in regulatory processes through the cyclin-dependent kinase pathway, preventing the activation of CDK kinases, which is related to senescence and apoptosis. Experimental research has linked *CDKN2A/B* genes to atherosclerotic plaque stability and the control of metabolism, particularly liver insulin resistance and adipose tissue plasticity [22]. These loci have been shown to influence diabetes and CVD risk across diverse study populations [22, 23] and to further elevate the risk of CAD in the presence of elevated glucose levels [24]. In a separate study including participants from the UK Biobank, a genetic variant near the *CCDC92* gene has been shown to influence both insulin resistance pathways and atherosclerosis [25], providing additional evidence that pathways related to insulin resistance could explain the links between the two diseases.

**Fig. 1:**
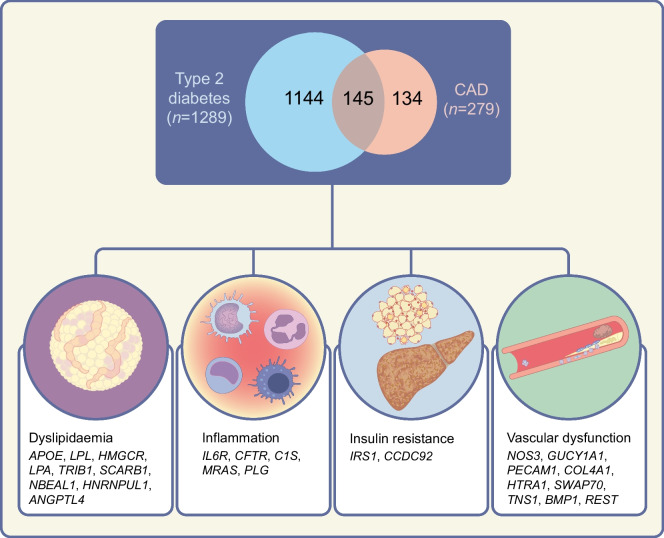
Shared genomic regions between type 2 diabetes and CAD. The number of shared variants identified from GWAS is illustrated in the Venn diagram. Type 2 diabetes GWAS from the Type 2 Diabetes Global Genetic Initiative (T2DGGI) and CAD GWAS from the Coronary ARtery DIsease Genome-wide Replication and Meta-analysis (CARDIoGRAM) plus The Coronary Artery Disease (C4D) Genetics (CARDIoGRAMplusC4D) consortium were analysed [14, 15]. Variants located within 500 kb of each other were considered to be shared between the two diseases. It is important to note that this method may overinterpret shared genomic regions and colocalisation analysis could further pinpoint the shared regions. The nearest genes of the shared variants were categorised according to the predicted mechanisms involved (dyslipidaemia, inflammation, insulin resistance or vascular dysfunction) based on previous studies [83, 84]. It is also important to note that the nearest genes are not necessarily the causal genes. This figure is available as part of a downloadable slideset

Advanced statistical genetic methods have been implemented to discover variants with concomitant effects in type 2 diabetes and CVD. One such approach is bivariate GWAS. A bivariate GWAS models the joint distribution of association with both outcomes, which helps improve the power to discover new loci for both outcomes simultaneously. In the largest bivariate GWAS for type 2 diabetes and CVD to date, there was evidence that 17 genomic regions overlap with the two diseases, which is more than expected by chance [26]. Among these signals was the *IRS1* region, again providing evidence of the relevance of insulin resistance pathways to the development of type 2 diabetes and CAD. The study also found directional consistency of the type 2 diabetes-related variants and risk of CAD broadly across the genome, suggesting that studies including larger sample sizes or more refined phenotypes can be critical to identifying common processes underlying both diseases. Figure 1 provides an overview of the shared genetic loci between type 2 diabetes and CAD and their related pathways. These loci are annotated based on the nearest gene, which is not necessarily the causal gene, and further functional studies are needed to interpret the roles of these variants in disease pathogenesis.

Another approach to identifying shared genetic variants between type 2 diabetes and cardiovascular traits is genome-wide interaction studies (GWIS) [27]. A GWIS is a methodological approach that tests whether a genetic variant is likely to affect the outcome of interest (i.e. CVD) only in the presence of a third variable (i.e. type 2 diabetes). Vujkovic et al found evidence of two genome-wide significant loci for type 2 diabetes-related CAD in a large-scale GWIS of European participants [28]; the study discovered a new variant near the *SORT1* gene (rs602633) and replicated the previous signal in the 9p21 region (rs1831733), which provides greater protection against CAD in individuals with type 2 diabetes than in those without type 2 diabetes. Sortilin, the protein product of the *SORT1* gene, functions as an intracellular sorting receptor for apolipoprotein B100. Genetic deletion of sortilin in mice reduces hepatic lipoprotein secretion, ameliorates hypercholesterolaemia and attenuates atherosclerotic lesion formation in LDL receptor-deficient animals [29]. Conversely, sortilin overexpression enhances hepatic lipoprotein release and elevates plasma LDL-C levels. The finding of stronger protection against CAD in the diabetic state might concur with the paradoxical association between lowering LDL-C levels and increasing type 2 diabetes risk [30].

An alternative way to identify genes and molecular mechanisms that contribute to both type 2 diabetes and CVD is by investigating temporal associations. Building on this concept, Kwak et al conducted a time-to-event GWAS in a large, multi-ethnic prospective cohort of people with type 2 diabetes who had not yet developed CVD at enrolment [31]. The analysis included 49,230 participants from the Cohorts for Heart and Aging Research in Genetic Epidemiology (CHARGE) Consortium, followed for a median of 10 years. The study identified three distinct genetic variants associated with an increased risk of incident CVD in individuals with type 2 diabetes (rs147138607 near *CACNA1E*/*ZNF648*, rs77142250 near *HS3ST1*, and rs335407 near *TFB1M*/*NOX3*). Interestingly, the rs147138607 variant was in close proximity to, although not in linkage disequilibrium with, the *GLUL* locus, which has been reported to be associated with CAD [32] and mortality [33] in people with type 2 diabetes. These three variants did not significantly influence CVD risk in the general population. Furthermore, the study found that individuals with type 2 diabetes were more likely to carry genetic variants previously linked to CAD. These findings illustrate how a principled approach that accounts for temporal relationships could lead to the discovery of novel loci associated with incident CVD in people with type 2 diabetes.

Given the potential variations in the underlying pathophysiology of type 2 diabetes and CVD across different populations, studies investigating the shared genetic architecture outside European ancestry are crucial. A recent study in a Chinese population identified a non-coding variant near *PDE1A* specifically associated with CAD risk in individuals with type 2 diabetes [34]. Notably, this variant interacted with BP control. Carriers of the protective allele for this variant paradoxically displayed a threefold increased risk for cardiovascular events in those with adequate BP control compared with those with uncontrolled BP. Conversely, individuals with the risk allele showed a significant 42% reduction in risk when their BP was adequately managed. A study in an Egyptian population revealed a 2.4-fold increased risk for CVD in individuals with type 2 diabetes carrying the *APOE* E3/E4 genotype [35]. A recent multi-ancestry GWAS of CAD demonstrated that the common haplotypes at the 9p21 locus were associated with CAD risk in all populations except in the African population, in which these haplotypes were absent [36]. These findings highlight the potential for population-specific genetic risk factors for type 2 diabetes-associated CVD. Further research is warranted to elucidate the broader landscape of shared genetic risk factors across diverse populations.

## Overlapping genetic signals from partitioned polygenic scores

In addition to genetic discovery approaches, polygenic scores generated from genetic variants associated with type 2 diabetes can be used to identify individuals with diabetes who are more likely to develop complications. Previous genetic studies have shown the effectiveness of polygenic scores in predicting type 2 diabetes risk and its related cardiovascular complications. Individuals with a higher genetic risk score for type 2 diabetes and/or its related traits were found to have a significantly greater chance of developing microvascular and macrovascular complications [28, 37]. However, traditional polygenic scores generated by simply aggregating genetic variants without considering the specific mechanisms by which these variants influence type 2 diabetes risk may be suboptimal for providing insights into the common molecular processes underlying diseases such as type 2 diabetes and CVD. Mechanistic information is needed to address the significant molecular heterogeneity of type 2 diabetes and its related complications.

In 2010, Voight et al attempted to understand the physiological role of type 2 diabetes genetic variants by examining their effects on insulin secretion (HOMA-B) and insulin resistance (HOMA-IR) [38]. Subsequently, Scott et al performed hierarchical hard clustering, identifying three main clusters of genetic variants based on their association patterns with diabetes-related traits, including BMI, lipid levels and glycaemic traits [39]. These include two clusters linked to insulin secretion and action, and one associated with obesity and dyslipidaemia. However, a key limitation of these clustering methods is the assumption that each variant solely influences a distinct, non-overlapping pathway. This is likely an oversimplification, as most genetic variants may contribute to multiple disease processes in different tissues or cell types.

More sophisticated soft clustering algorithms allow variants to influence multiple processes, better capturing the complex molecular heterogeneity underlying dysglycaemia. Udler et al applied a Bayesian soft clustering method to 94 type 2 diabetes variants and 47 diabetes-related traits [40]. This approach revealed five clusters of variants that increase the risk of type 2 diabetes as a result of specific pathophysiological processes. These included two clusters related to insulin deficiency due to either beta cell dysfunction or impaired proinsulin processing, and three clusters linked to insulin resistance, including obesity-mediated insulin resistance, adverse body fat distribution and disrupted liver lipid metabolism. The variants within each of the genetic clusters can be used to generate ‘partitioned’ polygenic scores that capture the genetic contribution to each intermediary process. The allocation of type 2 diabetes variants to each cluster is supported by tissue-specific patterns of chromatin accessibility, histone modification and transcriptional regulation, indicating that the mechanistic basis of these variants is robust even though these variants may have pleiotropic effects [40]. These partitioned polygenic scores showed that individuals whose diabetes risk is driven by adverse body fat distribution were at increased risk of CAD. This association was also observed in an independent analysis across a large-scale multicohort study including the CHARGE Consortium and the UK Biobank [41]. The implementation of this clustering approach within a multi-ethnic dataset led to the identification of additional type 2 diabetes genetic clusters that encompass a broader range of biological mechanisms [42]. This approach also provides preliminary insights into ancestry-related differences in type 2 diabetes risk profiles, helping to explain variations in genetic risk across diverse populations [42].

Recent genetic discoveries from the Type 2 Diabetes Global Genetic Initiative (T2DGGI) have further refined our understanding of the biological processes and molecular pathways contributing to type 2 diabetes and its complications [14]. This study nearly doubled the number of genetic variants associated with type 2 diabetes compared with previous efforts, and the new study consistently identified the previously reported five clusters of variants linked to specific pathophysiological processes [40]. Additionally, it highlighted three previously unreported clusters with cardiometabolic profiles representative of the metabolic syndrome, body fat and residual glycaemic effects. Individuals at increased genetic risk of type 2 diabetes due to variants in the obesity cluster had an increased risk of CAD and other complications. Similarly, genetic variants within the ‘beta cell + proinsulin’ cluster score were linked to a lower risk of CAD. These associations remained significant even after accounting for overall type 2 diabetes genetic risk. The relevance of the obesity cluster was confirmed in a separate prospective clinical trial, in which individuals with higher type 2 diabetes risk from pathways influencing obesity were at increased risk of heart failure [14]. Interestingly, the obesity cluster score was also associated with an earlier age at diabetes diagnosis, highlighting the importance of obesity-related processes in both the onset and the complications of type 2 diabetes.

**Fig. 2:**
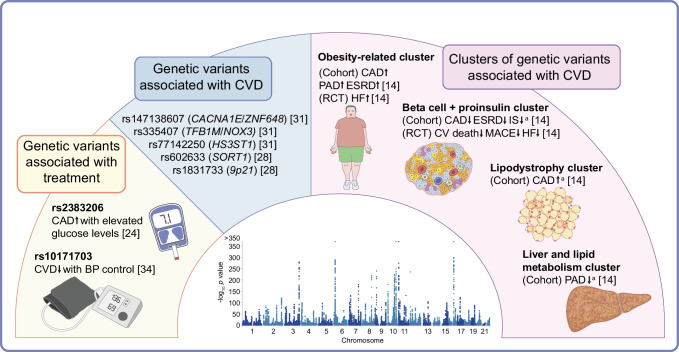
Precision risk evaluation of type 2 diabetes-related CVD using genetic information. The figure shows the genetic markers associated with CVD complications in people with type 2 diabetes. Markers on the left are associated with CVD in people with type 2 diabetes as single variants, and markers on the right are associated as clusters. ^a^Markers showing only a nominal association and that did not pass the Bonferroni-corrected significance threshold. CV, cardiovascular; ESRD, end-stage renal disease; HF, heart failure; IS, ischaemic stroke; MACE, major adverse cardiovascular event; PAD, peripheral artery disease. Manhattan plot reproduced from Suzuki et al [14] under the terms of the CC BY 4.0 license (http://creativecommons.org/licenses/by/4.0/). This figure is available as part of a downloadable slideset

This body of evidence underscores the advantages of partitioned polygenic scores over traditional type 2 diabetes polygenic scores, not only for identifying individuals at risk for specific complications but also for providing molecular insights into disease mechanisms and progression (Fig. 2). However, the predictive accuracy of current subtyping methods remains limited. Enhancing accuracy will require careful examination of contributing factors, including methodological constraints, data quality and sample size [43]. Addressing these limitations is essential for advancing the field and improving the clinical utility of these subtyping approaches.

## Causal evidence of molecular processes underlying CVD in type 2 diabetes

Targeted therapy to prevent CVD in individuals with type 2 diabetes should be grounded in causal risk factors. While it is commonly believed that type 2 diabetes leads to CVD, there may also be situations where CVD or declining heart function contributes to the development of type 2 diabetes, perhaps mediated by factors such as reduced physical activity or depression. However, Mendelian randomisation (MR) studies provide little evidence to support the notion that CVD is a causal risk factor for type 2 diabetes.

The causal role of dysglycaemia as a risk factor for CVD in type 2 diabetes has long been debated. While various epidemiological studies suggest that dysglycaemia is a key factor in the development of CVD in people with type 2 diabetes, clinical trials focusing on intensive glycaemic management to achieve near-normal blood glucose levels have not consistently demonstrated improvements in cardiovascular outcomes [44]. To address these discrepancies and better understand the causal relationships, researchers have used standard epidemiological methods such as MR [45]. MR can strengthen causal inference by using genetic variants as instrumental variables. MR studies suggest that type 2 diabetes and related glycaemic traits directly contribute to CVD development (Tables 1 and 2). The first MR study on this topic was published in 2015 and showed that elevated HbA_1c_ is causally associated with an increased risk of CAD, which is consistent with findings from large observational studies [46]. However, the high genetic correlation among traits makes identifying the main causal drivers challenging. An MR study led by Merino et al, which used genetic variants associated with hyperglycaemia without affecting other risk factors such as adiposity or lipids, supports a causal link between hyperglycaemia per se and CAD [47]. Further evidence comes from another MR on prediabetes, showing that early glycaemic alterations are likely to cause CAD, and that CAD prevention is likely to be most effective if initiated prior to the onset of diabetes [48]. Two genetic studies provided consistent evidence that the missense variants Ala316Thr and Arg131Gln in the glucagon-like peptide-1 receptor gene (*GLP1R*) are associated with lower fasting glucose and confer protection against CAD risk [49, 50].

**Table 1:** Summary of MR estimates of the effects of type 2 diabetes on CVD

Exposure	Outcome	First author (year)	Population of exposure (case/control)	Population of outcome (case/control)	SNPs	OR (95% CI)	*p* value	Increment
T2D	CAD	Gan (2019) [76]	DIAGRAM EUR (26,676/132,532)	CARDIoGRAMplusC4D EUR (60,801/123,504)	86	1.09 (1.04, 1.15)	9.32×10^−4^	Unit increase in log-odds of T2D
T2D	CAD	Wu (2024) [77]	DIAMANTE EUR (80,154/853,816)	FinnGen EUR (43,518/333,759)	153	1.14 (1.10, 1.19)	5.51×10^−11^	Unit increase in log-odds of T2D
T2D	CAD	Ruan (2024) [78]	DIAMANTE EUR (80,154/853,816)	FinnGen EUR (47,550/313,400)	145	1.12 (1.08, 1.16)	1.56×10^−9^	Unit increase in log-odds of T2D
T2D	CAD	Ruan (2024) [78]	DIAMANTE EUR (80,154/853,816)	CARDIoGRAMplusC4D + UKBB EUR (181,522/984,168)	141	1.10 (1.08, 1.13)	2.59×10^−16^	Unit increase in log-odds of T2D
T2D	IS	Wu (2024) [77]	DIAMANTE EUR (80,154/853,816)	FinnGen EUR (16,857/283,057)	152	1.13 (1.07, 1.19)	2.65×10^−6^	Unit increase in log-odds of T2D
T2D	IS	Ruan (2024) [78]	DIAMANTE EUR (80,154/853,816)	FinnGen EUR (25,398/339,920)	153	1.14 (1.11, 1.17)	3.52×10^−20^	Unit increase in log-odds of T2D
T2D	Stroke	Ruan (2024) [78]	DIAMANTE EUR (80,154/853,816)	GIGASTROKE EUR (73,652/1,234,808)	155	1.09 (1.06, 1.11)	1.02×10^−14^	Unit increase in log-odds of T2D
T2D	Stroke	Zhang (2023) [79]	DIAGRAM EUR (74,124/824,006)	GIGASTROKE EUR (73,652/1,234,808)	345	1.07 (1.06, 1.09)	3.44×10^−15^	Unit increase in log-odds of T2D
T2D	Stroke	Zhang (2023) [79]	AGEN-T2D GWAS EAS (77,418/356,122)	GIGASTROKE EAS (27,413/237,242)	181	1.03 (1.01, 1.06)	3.82×10^−3^	Unit increase in log-odds of T2D
T2D	PAD	Xiu (2022) [80]	UKBB EUR (21,926/342,747)	UKBB EUR (5674/359,551)	80	1.12 (1.06, 1.18)	6.87×10^−5^	Unit increase in log-odds of T2D
T2D	PAD	Xiu (2022) [80]	BBJ EAS (36,614/155,150)	BBJ EAS (3593/208,860)	94	1.18 (1.10, 1.27)	1.18×10^−6^	Unit increase in log-odds of T2D
T2D	PAD	Wu (2024) [77]	DIAMANTE EUR (80,154/853,816)	FinnGen EUR (11,924/288,638)	152	1.28 (1.20, 1.36)	1.41×10^−13^	Unit increase in log-odds of T2D
T2D	CKD	Zheng (2021) [81]	DIAMANTE EUR (80,154/853,816)	UKBB EUR (6985/454,323)	226	1.08 (1.05, 1.12)	1.76×10^−7^	Unit increase in log-odds of T2D
T2D	CKD	Zheng (2021) [81]	AGEN-T2D EAS (77,418/356,122)	BBJ EAS (8586/133,808)	142	1.07 (1.03, 1.10)	1.66×10^−4^	Unit increase in log-odds of T2D

Effect sizes are given as ORs (95% CIs)

AGEN-T2D, Asian Genetic Epidemiology Network Type 2 Diabetes Consortium; BBJ, Biobank Japan; CARDIoGRAMplusC4D, Coronary ARtery DIsease Genome-wide Replication and Meta-analysis (CARDIoGRAM) plus The Coronary Artery Disease (C4D) Genetics consortium; CKD, chronic kidney disease; DIAGRAM, DIAbetes Genetics Replication And Meta-analysis consortium; DIAMANTE, Diabetes Meta-Analysis of Trans-Ethnic association studies Consortium; EAS, East Asian; EUR, European; IS, ischaemic stroke; PAD, peripheral artery disease; T2D, type 2 diabetes; UKBB, UK Biobank

**Table 2:** Summary of MR estimates of the effects of glycaemic traits on CVD

Exposure	Outcome	First author (year)	Population of exposure (total participants)	Population of outcome (case/control)	SNPs	OR (95% CI)	*p* value	Increment
FG	CAD	Merino (2017) [47]	MAGIC EUR (up to 133,010)	CARDIoGRAMplusC4D EUR (63,746/130,681)	12	1.43 (1.14, 1.79)	0.002	1 mmol/l
FG	CAD	Yuan (2022) [51]	MAGIC EUR (up to 200,622)	CARDIoGRAMplusC4D mixed ancestry + FinnGen EUR (143,745/622,308)	71	1.18 (1.04, 1.33)	0.009	1 mmol/l
FG	CAD	Walker (2022) [82]	MAGIC EUR (up to 133,010)	CARDIoGRAMplusC4D mixed ancestry (60,801/123,504)	30	1.29 (1.06, 1.37)	0.013	SD units
2hGlu	CAD	Yuan (2022) [51]	MAGIC EUR (up to 63,396)	CARDIoGRAMplusC4D mixed ancestry + FinnGen EUR (143,745/622,308)	14	1.19 (1.07, 1.34)	0.002	1 unit in log-transformed mmol/l
FI	CAD	Yuan (2022) [51]	MAGIC EUR (up to 151,013)	CARDIoGRAMplusC4D mixed ancestry + FinnGen EUR (143,745/622,308)	38	1.88 (1.45, 2.44)	1.80×10^−6^	1 unit in log-transformed pmol/l
FI	CAD	Walker (2022) [82]	MAGIC EUR (up to 108,557)	CARDIoGRAMplusC4D mixed ancestry (60,801/123,504)	14	1.48 (1.20, 1.82)	0.036	SD units
HbA_1c_	CAD	Yuan (2022) [51]	MAGIC EUR (up to 146,806)	CARDIoGRAMplusC4D mixed ancestry + FinnGen EUR (143,745/622,308)	75	1.26 (1.04, 1.51)	0.016	1 percentage point
FG	IS	Yuan (2022) [51]	MAGIC EUR (up to 200,622)	MEGASTROKE + FinnGen + UKBB EUR (52,074/603,070)	71	1.15 (1.02, 1.29)	0.022	1 mmol/l
2hGlu	IS	Yuan (2022) [51]	MAGIC EUR (up to 63,396)	MEGASTROKE + FinnGen + UKBB EUR (52,074/603,070)	14	1.09 (0.99, 1.20)	0.070	1 unit in log-transformed mmol/l
FI	IS	Yuan (2022) [51]	MAGIC EUR (up to 151,013)	MEGASTROKE + FinnGen + UKBB EUR (52,074/603,070)	38	1.32 (1.05, 1.65)	0.016	1 unit in log-transformed pmol/l
HbA_1c_	IS	Yuan (2022) [51]	MAGIC EUR (up to 146,806)	MEGASTROKE + FinnGen + UKBB EUR (52,074/603,070)	75	1.01 (0.85, 1.21)	0.886	1 percentage point
FG	PAD	Yuan (2022) [51]	MAGIC EUR (up to 200,622)	FinnGen + UKBB EUR (12,032/569,149)	71	1.43 (1.16, 1.76)	0.001	1 mmol/l
FG	PAD	Walker (2022) [82]	MAGIC EUR (up to 133,010)	Million Veteran Program EUR (31,307/211,753)	30	2.26 (1.47, 2.40)	2.23×10^−4^	SD units
2hGlu	PAD	Yuan (2022) [51]	MAGIC EUR (up to 63,396)	FinnGen + UKBB EUR (12,032/569,149)	14	1.35 (1.12, 1.62)	0.002	1 unit in log-transformed mmol/l
FI	PAD	Yuan (2022) [51]	MAGIC EUR (up to 151,013)	FinnGen + UKBB EUR (12,032/569,149)	38	1.91 (1.25, 2.91)	0.003	1 unit in log-transformed pmol/l
FI	PAD	Walker (2022) [82]	MAGIC EUR (up to 108,557)	Million Veteran Program EUR (31,307/211,753)	14	2.51 (1.42, 2.67)	1.64×10^−3^	SD units
HbA_1c_	PAD	Yuan (2022) [51]	MAGIC EUR (up to 133,010)	FinnGen + UKBB EUR (12,032/569,149)	75	1.29 (0.95, 1.74)	0.100	1 percentage point

Effect sizes are given as ORs (95% CIs)

2hGlu, 2 h glucose after an oral glucose challenge; EUR, European; FG, fasting glucose; FI, fasting insulin; MAGIC, Meta-Analysis of Glucose and Insulin-related Traits Consortium; CARDIoGRAMplusC4D, Coronary ARtery DIsease Genome-wide Replication and Meta-analysis (CARDIoGRAM) plus The Coronary Artery Disease (C4D) Genetics consortium; UKBB, UK Biobank

The most comprehensive assessment to date of the causal effects of dysglycaemia on atherosclerotic and thrombotic events was published in 2022 [51]. This study leveraged publicly available genetic data for four glycaemic traits from the Meta-Analysis of Glucose and Insulin-related Traits Consortium (MAGIC)—fasting glucose, fasting insulin, 2 h glucose and HbA_1c_—and outcome data for 12 atherosclerotic and four thrombotic outcomes from the UK Biobank and FinnGen studies. The study provides evidence that dysglycaemia increases the risk of atherosclerotic outcomes but not thrombotic events. These findings align with experimental research showing that elevated blood sugar can trigger changes in blood vessel cells, promote inflammation and contribute to the pathogenesis of atherosclerosis [52]. Additionally, long-term exposure to hyperglycaemia leads to the formation of advanced glycation end-products, which accumulate in vascular tissues, thereby contributing to vascular stiffness and impaired vasodilation [53]. Thus, genetically determined hyperglycaemia is causally associated with CVD development and accelerates the deterioration of glucose homeostasis, highlighting the interconnected nature of type 2 diabetes and CVD [54].

In addition to hyperglycaemia, factors related to insulin resistance are crucial for understanding the links between dysglycaemia and cardiovascular complications. Using an integrative genomic approach, Lotta et al identified 53 genomic regions associated with insulin resistance phenotypes [55]. These regions were found to be linked to increased cardiometabolic risk, with the underlying mechanism involving an association with reduced adipose mass in peripheral compartments. More recent studies have employed dynamic insulin measurements to explore glucoregulatory mechanisms in skeletal muscle and adipose tissue, shedding further light on the relationship between peripheral insulin resistance and CVD [56]. Together, these findings support the relevance of insulin resistance in cardiometabolic health. However, the development of more accurate measures or proxies for insulin resistance will be essential to refine and deepen these insights at tissue and cellular levels.

Parallel to this, there is a paradoxical effect whereby lowering LDL-C reduces CVD risk but increases the risk of type 2 diabetes. Meta-analyses of RCTs have shown that medications designed to lower LDL-C, while demonstrably cardioprotective with lipid-lowering effects, may modestly increase the risk of type 2 diabetes [57]. Supporting this observation, individuals with specific genetic variations in genes targeted by LDL-C-lowering therapies (e.g. *HMGCR*, *NPC1L1*, *PCSK9*) exhibit impaired insulin sensitivity and a greater propensity to develop type 2 diabetes, particularly those with impaired glucose tolerance [58].

A noteworthy study by Klimentidis et al employed a two-stage GWAS design to identify 44 distinct genomic regions exhibiting opposing effects on LDL-C levels and type 2 diabetes risk [59]. These findings add further weight to the paradox that lowering LDL-C reduces CVD risk but increases the risk of type 2 diabetes. Analysis of the identified regions suggests enrichment for genes involved in hepatic fatty acid uptake and adipose tissue dysfunction, potentially linking the observed diabetogenic effects of LDL-C-lowering medications. This aligns with established knowledge from both animal and human studies demonstrating that hepatic fat accumulation can induce insulin resistance in the liver [60]. Additionally, these studies highlight the complex interplay between insulin's regulation of hepatic glucose and fat production, involving both direct and indirect mechanisms. A recent MR study, specifically designed to quantify the contribution of adiposity in this paradoxical association, provides further evidence that the diabetogenic effect attributed to LDL-C lowering may be partially mediated through increased adiposity [61].

While the cardioprotective benefits of lipid-lowering medications significantly outweigh the increased risk of type 2 diabetes, findings from these studies suggest that careful monitoring of body weight and glycaemic status might be prudent, especially for individuals at high risk for type 2 diabetes. In addition, the relevance of adipose tissue and liver to the diabetogenic effect of lowering LDL-C might support further investigations to better understand the molecular mechanisms by which lowering LDL-C might impact adipocyte function, lipid metabolism and dysglycaemia.

## Research gaps, opportunities and future directions

While current medical guidelines prioritise achieving optimal glycaemic control and managing cardiovascular risk factors to prevent CVD in individuals with type 2 diabetes, a more targeted approach rooted in the shared pathophysiology between type 2 diabetes and CVD may provide substantial benefits. Although recent advances in weight loss medications have demonstrated efficacy in reducing cardiovascular risk for millions globally, these therapies alone are insufficient to address the escalating cardiometabolic disease crisis. The high cost of these medications renders them inaccessible to vast populations and, even among those who can access them, therapeutic responses vary widely, highlighting that there is no universal treatment solution. To effectively prevent, diagnose and manage cardiometabolic diseases, we must address numerous unresolved questions about biological processes. This includes understanding the detailed behaviour and function of intracellular mechanisms, as well as the complex interactions between genetic predisposition and environmental factors involved in disease progression. What is critically needed is fundamental, interdisciplinary research that investigates the underlying mechanisms of cardiometabolic health outcomes across different life stages. Such insights will ultimately support precision health strategies tailored to the diverse needs of patient subgroups, contributing to more effective and equitable care.

How can we identify additional shared genetic loci between type 2 diabetes and CVD? Increasing sample sizes and including diverse populations are crucial to enhance power and capture causal variants across populations. Additionally, the development of more sophisticated statistical methods for colocalisation analysis and multi-trait fine-mapping could yield valuable insights. Beyond genetics, it is noteworthy that the expanding landscape of epigenomics, transcriptomics, proteomic and metabolomic studies is now being used to investigate the key signatures of type 2 diabetes subtype clusters and the shared features between type 2 diabetes and CVD [62, 63]. Both phenotypic and genotypic studies have consistently documented increased rates of cardiovascular complications among individuals with type 2 diabetes subtypes characterised by severe insulin resistance [40, 64]. A more refined understanding of these processes, particularly in the liver, adipose tissue and skeletal muscle, will be key to revealing the common drivers of both diseases and identifying strategies to advance precision diabetes medicine. Other environmental factors such as diet, physical activity, social determinants of health and in utero fetal programming should also be investigated.

While GWAS is a very useful starting point for identifying initial genomic regions associated with a given phenotype, much work is needed to depict the causal genes, cell types and mechanisms for which GWAS signals are relevant. Single-cell sequencing technology provides a powerful tool to unravel gene expression heterogeneity within individual cells, enabling a more nuanced understanding of GWAS signals. Single-cell transcriptomics and epigenetic profiling of human islets have identified *RFX6* as an important transcriptional factor in the early stages of type 2 diabetes pathophysiology [65]. This finding was supported by MR results from large-scale population datasets [65]. Innovative approaches that combine high-content imaging profiling with single-cell perturbation, such as the recently described LipocyteProfiler [66], are crucial for understanding deep molecular and cellular phenotypes that are shared by type 2 diabetes and CVD, as well as elucidating genetic effects on their heterogeneity. The use of classical cell lines, animal models and, more recently, human organoid models has provided valuable platforms for studying biological mechanisms [67]. Furthermore, genetic engineering using CRISPR technologies for either editing or genome-wide screening may shed light on variants’ effector genes and their biological function in relevant tissues [68–70].

Studies investigating the genetic overlap between type 2 diabetes and CVD have categorised type 2 diabetes into subclusters based on their shared pathophysiological processes, and these clusters have been linked with future cardiovascular complications. However, these studies have predominantly focused on European populations. Distinct type 2 diabetes subtypes observed in South Asian and other populations suggest that there are unique CVD risk profiles. For instance, Anjana et al identified two type 2 diabetes subgroups unique to a South Asian population characterised by features such as increased abdominal obesity and altered lipid profiles with different degrees of insulin resistance and deficiency: an insulin-resistant obese diabetes subtype and a combined insulin-resistant and insulin-deficient diabetes subtype [71]. These novel subtypes, which were not observed by Ahlqvist et al in the Scandinavian population [64], highlight the impact of race and ethnicity on the pathogenesis of type 2 diabetes and are particularly important as people in these clusters have increased rates of kidney disease and retinopathy. A recent subtyping approach in an Arab population also observed the five clusters identified by Ahlqvist et al [64], although the age at type 2 diabetes onset was younger than in the Scandinavian population, especially in the cluster related to mild obesity [72]. Investigating these race and ethnicity-specific subtypes may lead to the discovery of novel genetic risk factors underlying dysglycaemia and atherosclerosis and enable tailored interventions.

A recent study suggests that there is a stronger genetic link between early-onset type 2 diabetes and CVD [73]. The UK Biobank analysis revealed a higher genetic risk score for CAD in individuals diagnosed with type 2 diabetes at a younger age. Notably, the study found a 14% increase in genetically driven CAD risk for a 10-year earlier diagnosis of type 2 diabetes. A similar trend was observed in an East Asian hospital cohort, suggesting a potential link between early-onset type 2 diabetes and increased CVD risk influenced by a stronger genetic component [73]. Identifying the specific genes, molecular pathways and cellular mechanisms involved in these processes will be critical for developing targeted interventions for this vulnerable population. Research indicates that the high complication rate in early-onset type 2 diabetes is not solely attributable to a longer duration of dysglycaemia [74, 75]. Other factors such as the clustering of additional metabolic risk factors and challenging socioeconomic circumstances are also likely to play a role [9–11]. This is particularly relevant considering the increasing prevalence of multimorbidity, whereby young people with early-onset type 2 diabetes may experience irreversible microvascular damage and higher susceptibility to other diseases throughout their lives.

## Conclusions

This century has been marked by the growing challenge of diabetes and its associated cardiometabolic complications. While significant progress has been made in understanding the underlying mechanisms of both diseases, crucial knowledge gaps remain, particularly concerning the cellular and molecular processes that occur in the early stages of the disease and make people more prone to developing complications. Advancements in technology and sophisticated computational tools to identify directionality and identification of causal tissues with epigenomic and transcriptomic data provide exciting opportunities to bridge these gaps. By harnessing these tools, we can gain a deeper understanding of diabetes and cardiometabolic complications, paving the way for novel strategies to alleviate the burden of multimorbidity on individuals and society as a whole. Longitudinal analysis and RCTs are needed to generate solid evidence for precision medicine. At the societal level, mitigating the diabetes burden requires acknowledging the social determinants of health, such as access to healthy food and affordable quality healthcare, that significantly impact disease prevention and management. Future research and policy considerations must address these social factors, lifestyle modifications and genetic predisposition.

## Supplementary Information

Below is the link to the electronic supplementary material.

Slideset of figures (PPTX 614 KB)

## Abbreviations

ASCVD: Atherosclerotic CVD
CAD: Coronary artery disease
CHARGE: Cohorts for Heart and Aging Research in Genetic Epidemiology
GWAS: Genome-wide association study
GWIS: Genome-wide interaction study
LDL-C: LDL-cholesterol
MR: Mendelian randomisation
PAD: Peripheral artery disease
T2DGGI: Type 2 Diabetes Global Genetic Initiative
