# Supplementary material for: Exploring the shared genetic landscape of diabetes and cardiovascular disease: findings and future implications
Source: Diabetologia. 2025 Mar 15;68(6):1087–100. doi: 10.1007/s00125-025-06403-9 (PMC12069157; doi:10.1007/s00125-025-06403-9)
Supplement: Supplementary file 1 — Slideset of figures (PPTX 614 KB) [file 125_2025_6403_MOESM1_ESM.pptx]

## Slide 1
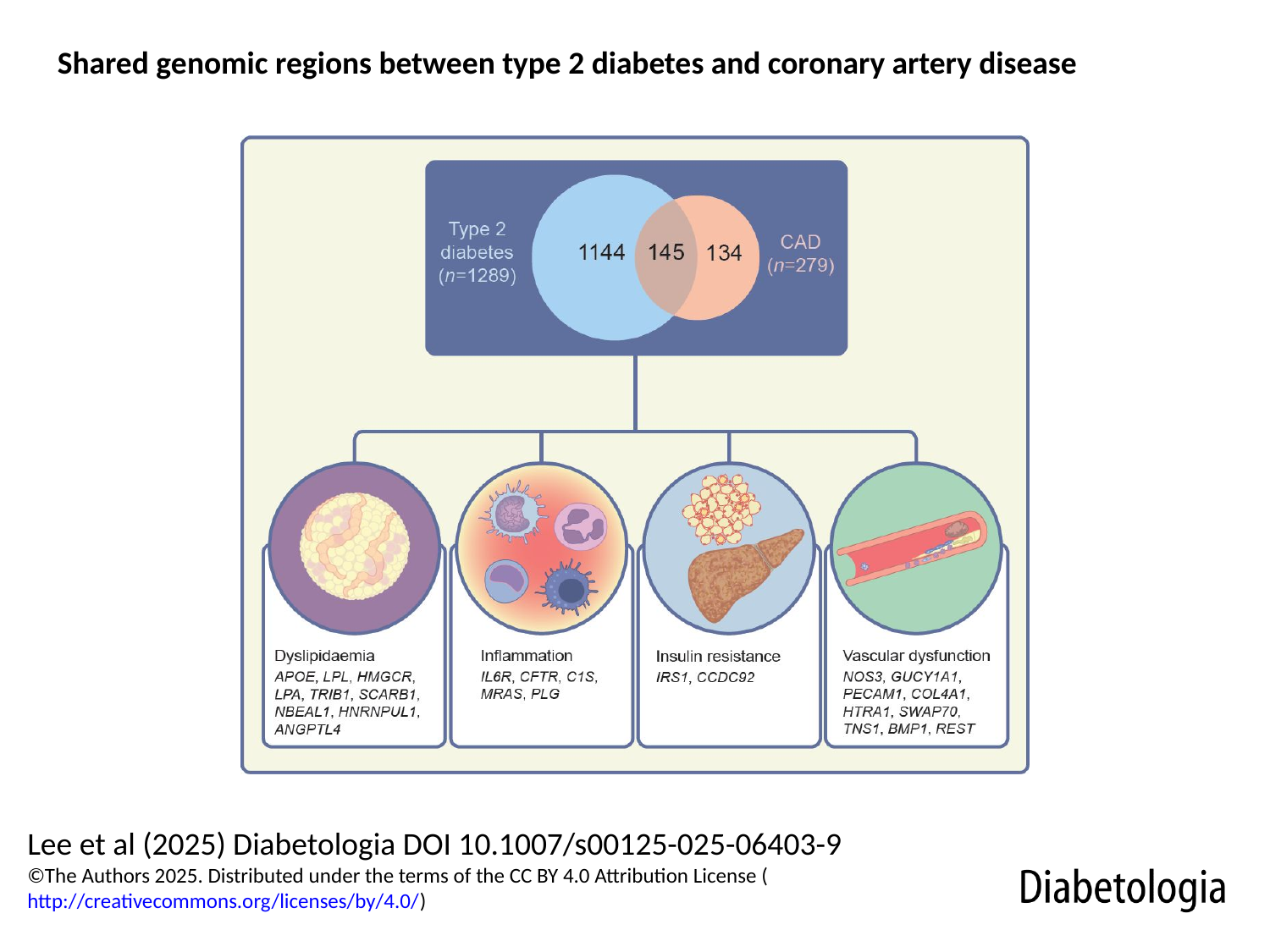

Shared genomic regions between type 2 diabetes and coronary artery disease
Lee et al (2025) Diabetologia DOI 10.1007/s00125-025-06403-9
©The Authors 2025. Distributed under the terms of the CC BY 4.0 Attribution License (http://creativecommons.org/licenses/by/4.0/)

## Slide 2
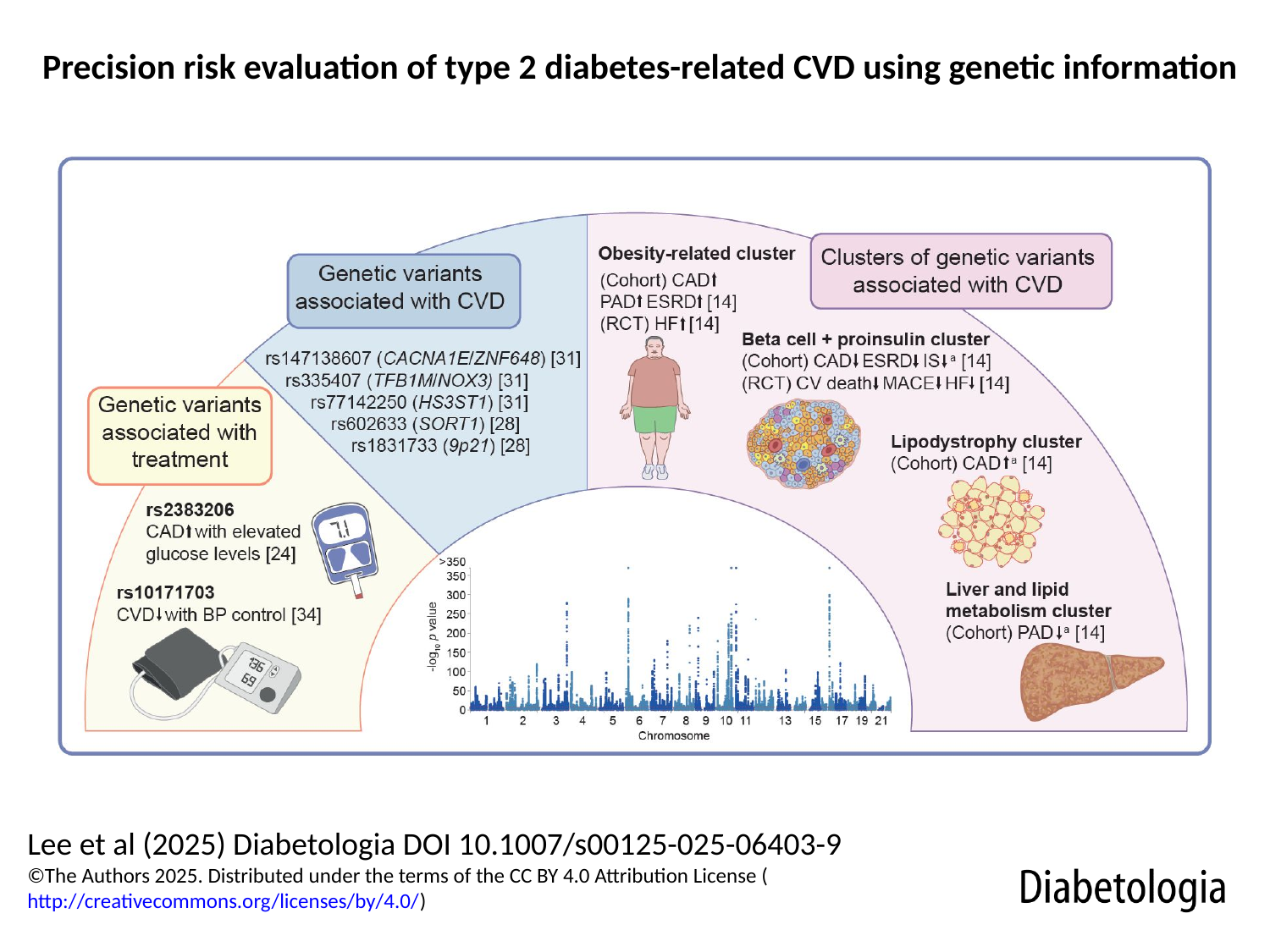

Precision risk evaluation of type 2 diabetes-related CVD using genetic information
Lee et al (2025) Diabetologia DOI 10.1007/s00125-025-06403-9
©The Authors 2025. Distributed under the terms of the CC BY 4.0 Attribution License (http://creativecommons.org/licenses/by/4.0/)
